# Late Enhancement Computed Tomography for Left Atrial Fibrosis Imaging: A Pilot “Proof-of-Concept” Study

**DOI:** 10.3390/diagnostics14232753

**Published:** 2024-12-06

**Authors:** Pietro G. Lacaita, Christoph Beyer, Fabian Plank, Markus Stühlinger, Gudrun M. Feuchtner

**Affiliations:** 1 Department of Radiology, Innsbruck Medical University, 6020 Innsbruck, Austria; placaita@gmail.com; 2 Department of Cardiology, Tyrol Clinicum Hall, 6060 Hall, Austria; christoph.beyer@i-med.ac.at (C.B.); fabian.plank@tirol-kliniken.at (F.P.); 3 Department of Cardiology, Innsbruck Medical University, 6020 Innsbruck, Austria; markus.stuehlinger@tirol-kliniken.at

**Keywords:** computed tomography, late enhancement, left atrial fibrosis, atrial fibrillation, left atrial catheter ablation

## Abstract

**Background/Objective**: Left atrial (LA) fibrosis imaging improves the guidance of LA catheter ablation. Cardiac computed tomography (CT) may be a reasonable alternative to CMR. The aim was to evaluate late enhancement (LE) fibrosis mapping by CT, and to correlate the results with low-voltage areas on electroanatomical mapping (EAM). **Methods**: In patients with atrial fibrillation who underwent 128-slice dual-source CT angiography (CTA) prior to LA catheter ablation, an additional LE-CT scan was performed 7 min after CTA. (1) Left atrial wall thickness (LAWT) was measured at three sites along the LA ridge. (2) Late enhancement (LE) was quantified co-axially aligned to LAWT and compared with low-voltage areas (LVA) on EAM. **Results**: Of 137 patients (age: 59.8 years; 27.7% females), 108 were included. The prevalence of LE was higher in patients with LAWT > 2 mm compared with 1.5 mm, with 78 (91.7%) vs. 77 (80.2%) (*p* = 0.022). Of 78 patients with LE, 60 (77.1%) had focal, 13 (16.5%) had diffuse, and 5 (6.3%) had mixed LE patterns. The CT density of focal LE was not different from that of diffuse patterns (104.2 +/− 21 HU vs. 98.9 +/− 18 HU; *p* = 0.360). Increasing LAWT and LE-HU were weakly correlated (r = 0.229; *p* = 0.041). LA wall artifacts had higher CT density compared with LE (154.1 HU vs. 114.2 HU; *p* = 0.002). The effective radiation dose was 0.95 mSv (range, 0.52–1.2 mSv) for LE-CT. The agreement of LE-CT was 80% for LVA < 0.5 mV and 86.6% for LVA < 0.7 mV in a subset of 30 patients. **Conclusions**: Left atrial fibrosis mapping by LE-CT is feasible. Late enhancement was found more frequently in LAWTs of more than 2 mm, and LE was correlated with increasing LA remodeling and low-voltage areas.

## 1. Introduction

Atrial fibrillation (AF) is the most common arrhythmia worldwide [1,2], associated with several severe conditions such as stroke, heart failure, and myocardial infarction, which significantly increase mortality rates [3,4].

Left atrial (LA) catheter ablation is one of the major therapeutic strategies for AF, targeting the restoration and the maintenance of sinus rhythm by ablating electrical triggers and drivers [5,6]. However, the recurrence rate can still exceed 40%. Several factors influence the persistence of AF, such as left atrial remodeling and antral pulmonary vein remodeling. Similarly, reduced left atrial strain, which is an indicator of left atrial fibrosis, has been recently linked to incident atrial fibrillation [7].

Left atrial fibrosis is a predictor of AF recurrence following successful ablation and plays a significant role in the progression from paroxysmal to persistent courses, which lead to unfavorable outcomes.

Indeed, the severity of LA fibrosis is associated with prior strokes, congestive heart failure (HF), diabetes, and arterial hypertension. Left atrial wall thickness (LAWT) quantified by computed tomography angiography (CTA) is a valuable surrogate marker for left atrial remodeling [8,9]. An increased LAWT predicts a higher recurrence rate of AF following LA ablation [10,11], as well as other cardiovascular outcome measures such as cryptogenic stroke [12].

Left atrial fibrosis has traditionally been quantified using a variety of techniques, such as two-dimensional speckle-tracking echocardiography (STE), late gadolinium enhancement magnetic resonance imaging (LGE-MRI), and voltage-based high-density electroanatomical mapping (EAM) [13]. However, EAM has limited ability to quantify and characterize the different types of fibrosis and it does not directly correlate with the precise amount or distribution of fibrosis. Further, subtle (mild or early) fibrosis may not result in significant electrical alternations detectable by EAM, since the spatial resolution may not be sufficient to identify small or minimal areas of fibrosis that could still contribute to the arrhythmia substrate. Furthermore, the quality of the electrical signals depends on the contact between the mapping catheter’s contact with the left atrial wall tissue.

Cardiac magnetic resonance imaging (CMR) with late gadolinium enhancement (LGE) is a more accurate approach for fibrosis detection due to its high accuracy [14,15] and improves the success rate of LA ablation [14]. Marrouche et al. (2014) [14] showed that patients with higher degrees of fibrosis quantified by CMR-LGE had significantly lower success rates and higher rates of AF recurrence, especially after several ablation procedures.

However, access to CMR is limited in certain clinical settings, such as in patients with MRI contraindications, and it is more difficult to perform in patients with arrhythmia. Furthermore, other non-invasive imaging techniques for LA function, such as STE, do not allow for direct imaging of fibrosis and instead provide an indirect estimate for LA fibrosis based on LA strain. Further, the accuracy of STE is dependent on the image quality, which can be compromised in patients with arrythmia or poor acoustic windows.

Performing an additional late enhancement (LE) CT scan after CTA for pulmonary vein imaging could enable the imaging of left atrial fibrosis. LE-CT could serve as an alternative to CMR, offering similar diagnostic capabilities with the added advantage of greater availability, faster acquisition times, and a higher spatial resolution. This could make LE-CT especially useful for selecting candidates who are more likely to benefit from LA ablation.

Therefore, the aim of our “proof-of-concept” study was to investigate if left atrial fibrosis mapping by LE-CT after CTA is feasible, aiming to compare LE-CT with LAWT, the established CT imaging marker for LA remodeling, to quantify LE and to describe LE patterns, and to compare them with low-voltage areas (LVA) on EAM.

## 2. Material and Methods

### 2.1. Study Design and Population

Patients with paroxysmal or persistent atrial fibrillation (AF) referred for ECG-synchronized thoracic CT angiography between 2015 and 2019 for planning clinically indicated left atrial (LA) catheter ablation [2] were included in our retrospective study registry. Institutional review board (IRB) approval for the database was obtained.

### 2.2. Computed Tomography (CT)

A 2-step scan protocol was applied.

Scan I. Thoracic computed tomography angiography (CTA) was performed with a 128-slice dual-source CT scanner (Somatom Definition FLASH, Siemens or DRIVE, Siemens Healthineers, Erlangen, Germany), a detector collimation of 2 × 64 × 0.6 mm, a z-flying spot, and a rotation time 0.28 s. The tube voltage was adapted to the patients’ size (80–120 kVp). Prospective ECG synchronization was applied, and the scan was triggered into the diastolic phase (70% of the RR interval) in high-pitch 3.2 (“Flash”) mode. The scan range covered the entire thoracic field for the purpose of pulmonary vein imaging. An iodine contrast agent (Iopromide, Ultravist 370™, Bayer Vital GmbH, Leverkusen, Germany) was injected intravenously (a flow rate of 4–6 mL/s, followed by 40 cc of a saline solution), triggering the arterial phase (bolus tracking; 100 HU threshold; ascending aorta). The contrast volume ranged from 65 cc up to 120 cc, depending on body weight, using a standardized regimen. Axial images were reconstructed with a 0.75 mm slice width (increments of 0.4 mm) and a medium-smooth kernel B26f).

Scan II. A non-contrast ECG-gated late enhancement (LE) CT scan with standardized scan parameters (detector collimation, 2 × 64 × 0.6 mm; rotation time, 0.28 s; prospective ECG synchronization, trigger into the diastolic phase) was performed in high-pitch mode (pitch factor, 3.4) 7 min after the CTA. The tube voltage was 80 kVp or 100 kVp. Axial images were reconstructed with a 0.75 mm slice width (increment 0.4/medium-smooth kernel B26f). The scan range covered the entire heart from the pulmonary bifurcation to the diaphragm.

CTA image analysis: Clinically approved radiology software (DeepUnity Diagnostic 1.2.0.1, Dedalus, DH Healthcare, Bonn, Germany) was used. The following measures were taken.

Scan I CTA: Left atrial wall thickness (LAWT) was measured at 3 sites along the anterior wall of the left atrium with a digital caliper, along the LA ridge. First, the region with maximal left atrial wall thickening was identified. Then two other adjacent sites with LAWT were selected, and LAWT was measured in order to cover the entire range of LAWT. The mean of the three measurements was calculated. LAWT was defined as a thickening of more than 1 mm.

**Figure 1:**
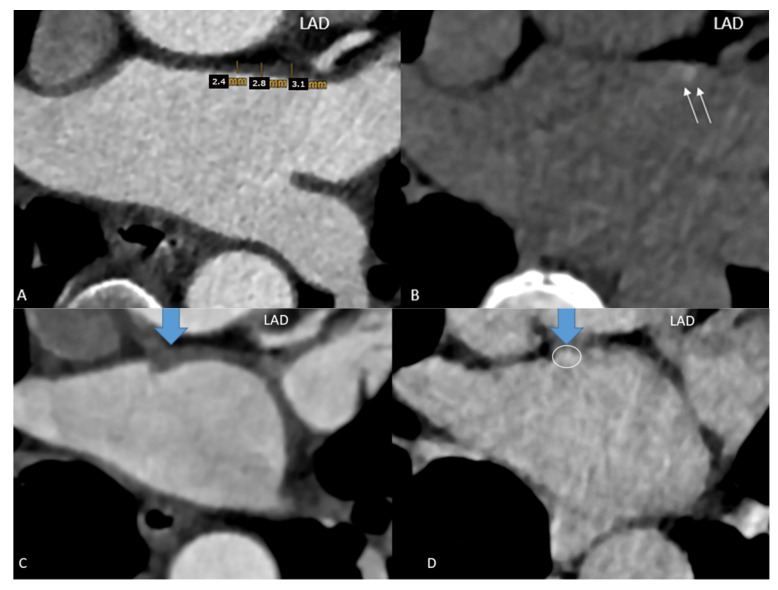
**Focal late enhancement** (**LE**)**.** Images (**A**) of the early phase CTA were co-axially aligned with late enhancement CT performed 7 min after contrast agent injection (**B**) (2-step CT protocol). (**A**) LAWT was measured at 3 sites along the LA ridge, and the mean was calculated. The left anterior descending (LAD) coronary artery served as a landmark for co-axial alignment in this patient: a 52-year-old-female with persistent AF, a CVRF of active smoking. The LAWT was >2 mm (**A**) and focal LE with 130 HU (**B**) was observed. (**C**,**D**) Results in a 54-year-old male with AF prior to planning LA ablation. The mean LAWT was 4 mm (**C**), and a focal dense spot of late enhancement (LE) was identified at the exact site of maximal LAWT, with 102 HU (**D**).

Scan II LE-CT: Axial LE-CT images were co-axially aligned with the CTA datasets on two screens. The left main and proximal left anterior descending (LAD) coronary arteries served as landmarks for co-axial image alignment (Figure 1).

LE was quantified by placing a round-shaped region of interest (ROI) into the co-axially aligned images into the areas of LAWT on CTA, in which either focal or diffuse hyperdense lesions were visible, and the CT density (Hounsfield units = HU) was measured. LE was defined as an enhancement of more than 90 HU. Focal and diffuse LE and mixed LE patterns were distinguished on a 3-point scale (1 = focal, 2 = diffuse linear, and 3 = mixed (both focal spots and linear patterns)). If there was more than 1 focal spot or a larger linear region with LE, all areas were measured, and the mean was calculated.

Left atrial wall artifacts (such as beam hardening, streaks, or motion artifacts) were recorded, distinguished subjectively from LE, and quantified by placing a round-shaped ROI.

Images with the following artifacts were excluded from LE quantification: 1 = motion blurring; 2 = beam hardening artifacts such as dark bands and/or streaks, caused by compromised energy from X-ray photons after passing through dense structures (with high CT attenuation values) such as bones or metal, and appearing as hypoattenuating bands and/or adjacent hyperattenuating streaks after a dense structure.

Coronary CTA analysis was performed by 2 experienced radiologists independently (one with >3 years and one with >10 years of training), who performed all LAWT and LE measurements. Interobserver variability was calculated. Second, one observer repeated all measurements after a minimum of 3 months.

Radiation dose: the effective radiation dose (mSv) was calculated from automated dose–length product (DLP) report values derived from the CT scanner, multiplied by the cardiothoracic specific conversion factor of k = 0.017.

### 2.3. Electroanatomical Mapping and Left Atrial Catheter Ablation

Pulmonary vein isolation without additional lines in the left atrium was performed in all patients by irrigated tip catheter radiofrequency ablation, with power settings between 30 and 45 W and the duration at the discretion of the treating electrophysiologist, depending on the patients’ individual characteristics. Radiofrequency ablations were exclusively conducted using a wide-area circumferential pulmonary vein isolation technique [10].

Electroanatomical (EA) bipolar voltage mapping (EAM) of the LA was performed at the beginning of each procedure, using the CARTO 3 system (Biosense Webster Inc., Irvine, CA, USA). All voltage maps were acquired using CARTO Pentaray catheters. A “low-voltage area” (LVA) was defined as an area with bipolar signal amplitudes of <0.5 mV in the sinus rhythm. All voltage maps with adequate area covering of the anterior and posterior LA wall (i.e., at least 500 points) were included. Within the entire anterior wall, low-voltage areas were localized visually, and 20 measure points in total were placed into low-voltage areas, radiating laterally. Co-axial anatomic alignment with LE-CT images was performed, and all low-voltage marker points were correlated with areas that were positive for LE by CT.

### 2.4. Statistical Analysis 

Statistical analysis was performed using SPSS™ software (V25.0, SPSS Inc., Chicago, IL, USA). Quantitative variables are expressed as means ± standard deviation (SD) and categorical variables as absolute values and percentages. Chi-square and Fisher’s exact tests were applied to test for differences in categorical data (the prevalence of LE in groups of patients with ≥2 mm and ≥1.5 mm LAWT cut-offs). Pearson’s correlation coefficient was calculated to define the correlation between LAWT and LE density (HU). The independent *t*-test was applied to test for differences in the CT density (HU) of LE and artifacts. Interobserver and intraobserver variability (reliability) was defined by the weighted Cohen’s Kappa, and additionally, for parametric data (mean LAWT), Pearson’s correlation coefficient was calculated, and Bland–Altman plots were generated.

## 3. Results

**Figure 2:**
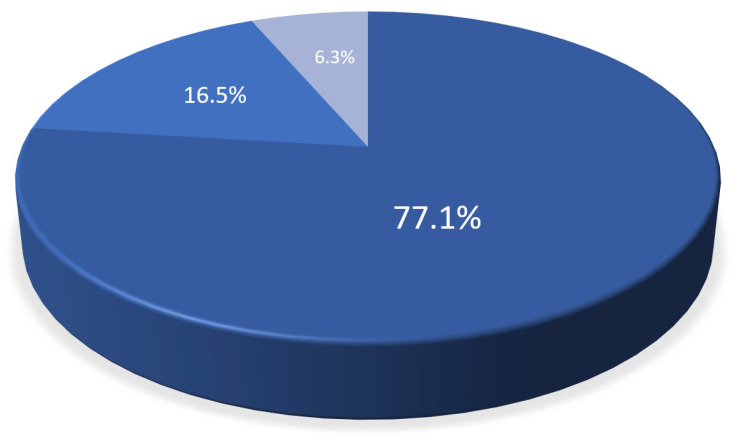
**Late enhancement** (**LE**) **patterns of left atrial fibrosis by CT.** The majority had focal (77.1%) LE, while diffuse (16.5%) and mixed LE patterns (*n* = 5, 6.3%) were less frequently observed.

**Figure 3:**
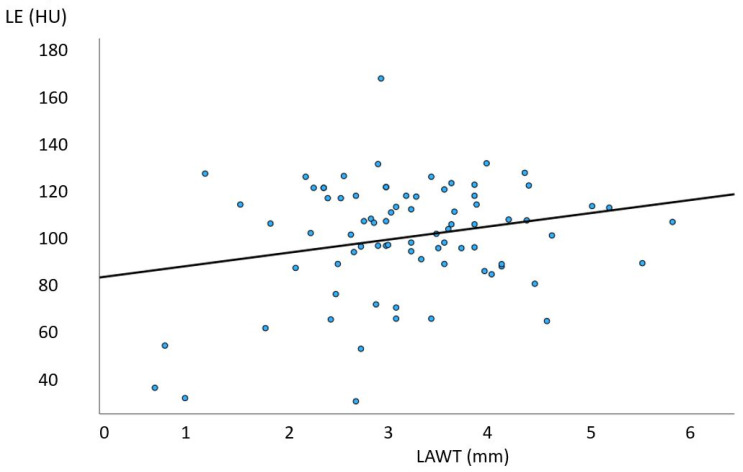
**Correlation of anterior left atrial wall thickness (LAWT) with late enhancement (LE) CT density (HU)**. A weak correlation was found (r = 0.229; *p* = 0.041). HU, Hounsfield units; mm, millimeters.

**Figure 4:**
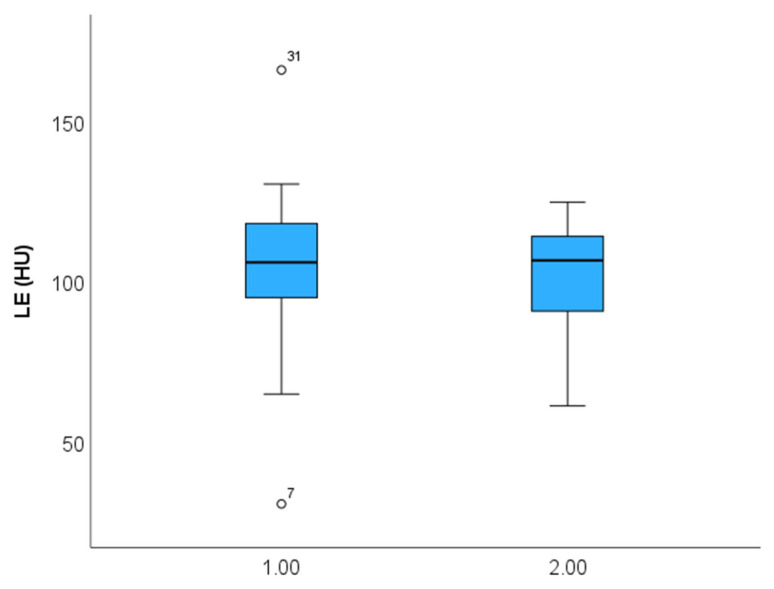
**Late enhancement** (**LE**) **density (HU)** in focal (1) compared with diffuse (2) fibrosis patterns. No difference was observed in CT density (Hounsfield Units, HU) (*p* = 0.360).

Of 137 patients (age: 59.8 years, 27.7% females), 108 CT scans were included. The main characteristics of the study cohort are summarized in Table 1. Of these, 98 had (91.5%) with LAWT > 1.5 mm and 84 (78.5%) had LAWT > 2 mm, and the remaining (8.5%) had LAWT between 1.0 and 1.5 mm. The prevalence of LE was higher in LAWT > 2 mm compared with LAWT > 1.5 mm with 78/98 (91.7%) vs. 77/84 (80.2%) (*p* = 0.022). Of 78 patients with LE, 60 (77.1%) had focal, 13 (16.5%) had diffuse, and 5 (6.3%) had mixed LE patterns. The total distribution of LE patterns is shown in Figure 2. The CT density of focal LE was not different from that of diffuse patterns (104.2 ± 21 HU vs. 98.9 ± 18 HU; *p* = 0.360) (Figure 3). Increasing LAWT and LE-HU were weakly correlated (r = 0.229; *p* = 0.041) (Figure 4). The mean LAWT was 2.67 +/−1.1 mm (maximal: 5.7 mm).

Left atrial wall artifacts, mainly attributable to beam hardening, streaks, and motion artifacts, showed higher CT attenuation values (mean: 154.11 HU) compared with LE (114.2 HU) (*p* = 0.002). The mean effective radiation dose for the LE-CT scans was 0.95 mSv (range: 0.52–1.2 mSv).

**Figure 5:**
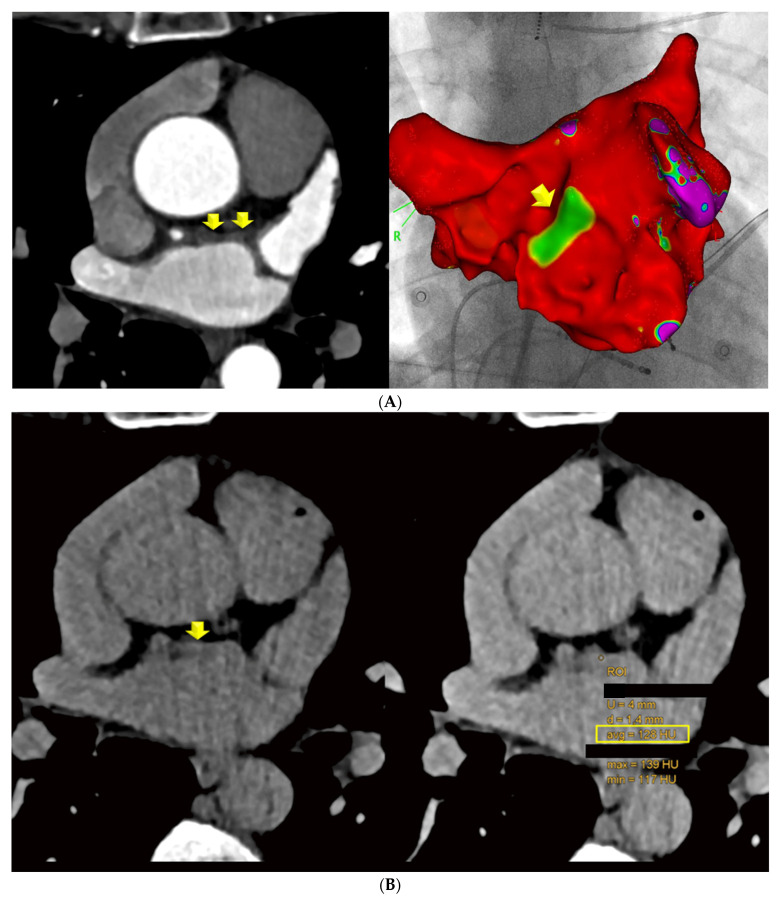
A 59-year-old-male with paroxysmal AF, a BMI of 21.5, the CVRF of dyslipidemia, and left atrial wall thickness (LAWT) of 3.3 mm, with a low-voltage area on EAM (anterior LA wall, green/yellow area) (upper panel, (**A**)) matching with focal LE (128 HU) on late enhancement (LE) CT (lower panel, (**B**)).

**Figure 6:**
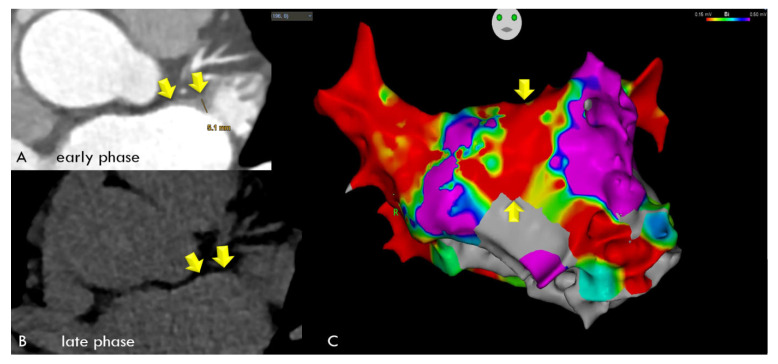
A 59-year-old male with a BMI of 27.8 kg/m^2^, and a LVEF of 68% with paroxysmal AFIB and severe left atrial wall thickening (LAWT) with a maximum of 5.1 mm during early-phase CT (Panel (**A**), arrows) and linear late enhancement during late-phase CT (Panel (**B**), arrows), indicating diffuse fibrosis, which was correlated with low-voltage areas along the entire anterior left atrial wall during electroanatomical mapping (EAM). (Panel (**C**)) All 20 spots were positive for low-voltage areas of <0.5 mV, shown as red areas (yellow arrows), with values ranging from 0.015 to 0.12 (mean: 0.024 mV). While LA ablation with pulmonary vein isolation was successful, the patient experienced recurrent AFIB after 3 months, and a second LA ablation was performed 7 months later.

In the comparison of LE-CT with the EA voltage maps, voltage maps were available in a subset of 30 patients. In the per-patient-based analysis, there were 24 agreements (80%) between LE-CT and the voltage maps (21 true positive and 3 true negative) for low-voltage areas (bipolar signals < 0.5 mV). There were six mismatches. In two patients with LE by CT, borderline low-voltage areas were present (0.67 and 0.69 mV); in two patients, LAWT (2.1 mm) was mild; and in two patients, the reasons were unclear (possible artifacts). Agreement was higher, with 28/30 (86.6%) for low-voltage areas <0.7 mV (Figure 5 and Figure 6). Figure 5 shows an example of a 59-year-old-male with paroxysmal AF, left atrial remodeling (LAWT 3.3 mm), and LE with 128 HU on the anterior wall correlating with a low-voltage area on EAM. Figure 6 shows a case with linear LE indicating diffuse fibrosis, with LVA and recurrent AFIB after ablation.

Intraobserver agreement was good, with kappa 0.832 (95% CI: 0.787–0.877, *p* < 0.001) for LAWT, and interobserver agreement was slightly lower, with kappa 0.743 (95% CI: 0.647–0.812, *p* < 0.001). The intraobserver correlation for mean LAWT was high, with r = 0.972 (95% CI: 0.957–0.982), and the interobserver correlation was r = 0.876 (95% CI: 0.812–0.919).

Bland–Altman plots (Figure S1) showed that for intraobserver agreement, the mean error for mean LAWT (average of three measurements) was minimal, with +0.0027 (95% CI: −0.0504–0.05598) and narrow limits of agreement (lower limit: −0.4661; upper limit: 0.4716). For interobserver agreement, the mean error was +0.07 (95% CI: −0.0286–0.1736), with narrow limits of agreement (lower limit: −0.8182; upper limit: 0.9632). For detection of LE, interobserver agreement was good, with kappa 0.895 (95% CI: 0.826–964, *p* < 0.001).

## 4. Discussion

In the past decade, late gadolinium enhancement (LGE) imaging using cardiac magnetic resonance imaging (CMR) has improved the detection of myocardial fibrosis [16] and has been demonstrated to be clinically relevant in patients with diabetes [17]. Late enhancement using CMR is also beneficial for left atrial fibrosis (LAF) mapping. Indeed, several studies have found an association between areas with left atrial LGE on CMR and low-voltage areas on electroanatomic mapping (EAM) [18]. However, in clinical practice, the LGE approach via CMR demands sophisticated protocols and also substantial resources due to the time-consuming nature of capturing images of thin atrial walls, and faces other technical challenges such as artifacts from irregular heartbeats [19]. Therefore, our explorative “proof-of-concept” study aimed to develop an accessible and faster alternative method for left atrial fibrosis imaging. Cardiac CT offers a superior spatial resolution to CMR and may improve the visualization of LA fibrosis. Because of its multiplanar reconstruction capabilities, cardiac CT has emerged as the preferred modality for advanced imaging of complex and thin structures, and provides accurate measurements in patients with AF, such as those used for planning LA ablation [20]. Our pilot study shows, for the very first time, that imaging of left atrial fibrosis with LE-CT is technically feasible by performing a delayed scan 7 min after CT angiography, as recommended for left ventricular myocardial late enhancement [21]. First and foremost, our study revealed a high prevalence of LA fibrosis in patients with increased left atrial wall thickness (LAWT) of more than 2 mm, as well as a correlation of LA fibrosis density with the amount of LAWT. Further, and most importantly, the majority of LE areas correlated well with bipolar low-voltage areas <0.5 V in patients undergoing LA ablation. Electroanatomical mapping (EAM) is considered as a validation tool for areas containing left atrial fibrosis. Prior studies have shown that LE imaging via CMR has a good agreement with EAM, although inconsistencies in image quality have been reported, leading to heterogenicity in a metanalysis [22]. Second, we identified two different patterns of fibrosis: focal dense spots and diffuse fibrosis (Figure 1, Figure 5 and Figure 6), which occurred in the majority. In a minority of patients, both patterns were present, consistent with existing literature highlighting fibrosis as a significant aspect of LA remodeling in patients with AF [23]. Nakamura et al.’s [8] study demonstrated a correlation between increased LAWT on CT angiography (CTA) and identified LAWT as valuable imaging biomarker for LA remodeling. However, to the best of our knowledge, no study to date has investigated whether it is possible to visualize fibrosis by late enhancement (LE), and no study yet has examined the correlation with LAWT. The identification of various patterns of late enhancement (focal, diffuse, and mixed) offers more insights into the heterogeneity of fibrotic remodeling in AF patients. Focal late enhancement was the most commonly observed pattern, suggesting localized areas of fibrosis, while diffuse late enhancement, though less frequent, indicated more widespread fibrotic changes. Artifacts such as motion blurring or beam hardening pose a significant challenge for the quantification of LA fibrosis by CT. Therefore, we identified artifacts, quantified by HU, and excluded them from the LE-CT analysis. Notably, artifacts had consistently higher HUs than LE-CT, highlighting the necessity of sophisticated image processing techniques and artifact correction software, which are pivotal in differentiating true fibrotic changes from imaging artifacts. This distinction is critical for ensuring that clinical decisions based on these scans, such as the planning of ablation procedures, are supported by precise and reliable data. Of note, distinguishing artifacts requires a high level of CT image interpretation experience. To ensure high-quality readouts in our study, both readers were qualified radiologists, and one had more than 10 years of CT expertise. Further, interobserver agreement was good, and intraobserver agreement was slightly higher. The LAWT measurements showed high reliability, while the interobserver agreement for LE was lower but still good. Finally, the radiation exposure of the appended LE-CT scan was minimized by applying a low-dose scan protocol with a low tube voltage (80–100 kV) and a high pitch factor (“flash mode”), resulting in consistent values of less than 1.2 mSv. Further, a low tube voltage is beneficial for optimal soft tissue contrast of the left atrial wall and thus for the detection of LE. Additionally, other potential factors for the recurrence of atrial fibrillation after successful ablation have already been identified in the CT scans, namely enhanced attenuation of posterior left atrial adipose tissue, which indicates local inflammation and has been associated with a higher risk of AF recurrence [24]. Thus, late enhancement may provide valuable information when selecting patients for AF ablation.

Our study has some limitations. The study population’s sample size is rather small, and we acknowledge the retrospective study design with its potential biases. Further research in larger cohorts is required to define the accuracy of LE. Second, artifacts mimicking LE pose a significant challenge and require a high level of expertise in CT interpretation for a visual distinction and may contribute to the moderate agreement of 80% (patient-based) between LE and EAM.

## 5. Conclusions

Our study shows that left atrial fibrosis mapping by LE-CT is technically feasible. Late enhancement was found more frequently in patients with a higher degree of LA remodeling (LAWT more than 2 mm), and LE density was correlated with increasing LAWT and well as with areas of low voltage in the corresponding electroanatomical maps. Our study contributes to the evolving landscape of atrial fibrillation management by underscoring the potential of LE-CT as a potentially valuable tool in the detection of left atrial fibrosis. It encourages further investigations, for example, to validate LE-CT in larger cohorts, in comparison with CMR, and for prediction of clinical outcomes, such as the recurrence rate of AF after LA ablation. Similar to CMR, fibrosis mapping by CT may enhance the precision of left atrial ablation planning, because it can help to select patients with AF who respond better to left atrial catheter ablation. Those patients with no or minimal LA fibrosis are more likely to restore and maintain sinus rhythm after LA ablation, while those with a high fibrosis content will instead suffer from recurrent AF. Areas with left atrial fibrosis represent non-viable tissue that is refractory to electrical signals. Fibrosis disrupts electrical propagation or slows down conduction because it forms barriers that prevent normal electrical signals from spreading across the atrial myocardium. Accordingly, fibrotic tissue is less responsive to antiarrhythmic drugs and has been associated with lower success rates after LA catheter ablation.

Current gaps in knowledge: Although EAM is useful for identifying areas of low voltage that may be associated with fibrosis, other conditions such as edema or inflammation may also slow down electrical conduction. Therefore, it is not yet clear how well EAM can predict outcomes such as the recurrence of atrial fibrillation or success after ablation. However, current scientific evidence indicates that CMR imaging of fibrosis [14,15] is a better and more specific surrogate marker for predicting outcomes after LA ablation. However, comparative studies of LE-CT with CMR would be desirable.

In the light of the recent introduction of a new scanner technology, photon-counting detector CT [25], which provides improved technical features (higher spatial resolution, ultrahigh-resolution (UHR) mode, and spectral imaging), LA fibrosis imaging may benefit as well.

Clinical perspective: Image-guided planning of LA catheter ablation assisted by left atrial fibrosis mapping via LE-CT is feasible. Left atrial fibrosis mapping by CT may provide an alternative to CMR for both the guidance of more precise LA ablation and as a prognosticator for outcomes in patients with atrial fibrillation; however further research, including validation studies in larger cohorts, is required. Future studies are needed to investigate the long-term clinical outcomes associated with LE-CT-guided ablation and to explore the cost-effectiveness and accessibility of LE-CT as an alternative to CMR.

## Figures and Tables

**Table 1:** Study cohort, *n* = 137.

Age (years)	59.8 +/− 9.6
Females	38 (27.7%)
BMI kg/cm^2^	26.8 +/− 4.2
**CVRF**	
Smokers (active)	14 (10.2%)
Arterial hypertension	50 (36.9%)
Dyslipidemia	56 (40.4%)
Diabetes	8 (5.8%)
**Atrial fibrillation type**	
Paroxysmal Persistent	91 (66.4%) 46 (33.6%)
**Medication**	
Vit-K inhibitors NOAC No anticoagulants (e.g., ASA)	9 (6.5%) 87 (63.6%) 41 (29.9%)

Abbreviations: BMI, body mass index; CVRF, cardiovascular risk factors; NOAK, novel oral anticoagulants (rivaroxaban, dabigatran, apixaban); ASA, acetylsalicylic acid.

## Data Availability

Upon request from the corresponding author.

## Supplementary Materials

The following supporting information can be downloaded at https://www.mdpi.com/article/10.3390/diagnostics14232753/s1, Figure S1: Bland Altman Plots showed a high agreement for both interobserver and intraobserver measurements of mean left atrial wall thickness (LAWT).
