# Supplementary material for: Late Enhancement Computed Tomography for Left Atrial Fibrosis Imaging: A Pilot “Proof-of-Concept” Study
Source: Diagnostics (Basel). 2024 Dec 6;14(23):2753. doi: 10.3390/diagnostics14232753 (PMC11640274; doi:10.3390/diagnostics14232753)

Figure S1: Bland Altman Plots showed a high agreement for both interobserver and intraobserver measurements of mean left atrial wall thickness (LAWT).

For intraobserver agreement, mean error was minimal with +0.003 (95% CI: -0.050 - 0.056) with narrow limits of agreement (lower limit: -0.466, and upper limit: 0.472).

For interobserver agreement, a mean error was low with + 0.07 (95 CI: -0.029- 0.174) and the limits of agreement were narrow (lower limit: -0.818, and upper limit: 0.963). SD = standard deviation.

Intraobserver agreement for LAWT:

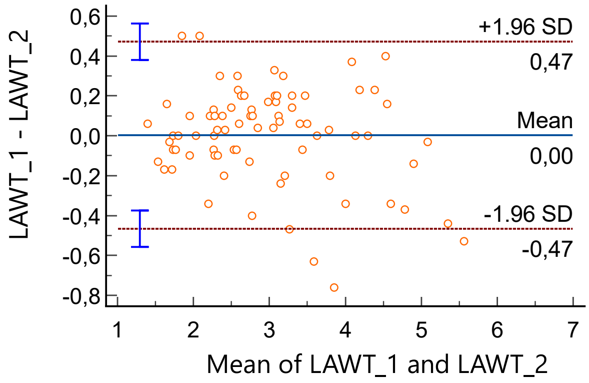

Interobserver agreement for LAWT: 01 = 1st Observer and 02 = 2nd Observer

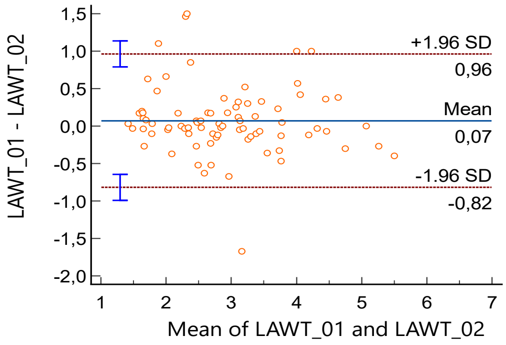

Supplement: Supplementary file 1 [file diagnostics-14-02753-s001.zip › diagnostics-3313542-supplementary.pdf]
